# The Art of Being Normal

**DOI:** 10.1192/pb.bp.115.051730

**Published:** 2016-12

**Authors:** Jennie Elizabeth Higgs

Recently there has been a wealth of creative work featuring transgender people on stage, page and screen. Add to that stories of transition from public figures and the rise in transgender people modelling for well-known fashion houses, and it becomes clear that important discussions about transgender issues are finding their place in the public consciousness.

Lisa Williamson's excellent debut novel *The Art of Being Normal* skilfully blends the difficulties of being a teenager with the social challenges of identifying as transgender. In the opening pages we meet David, a 14-year-old with a secret. He tells us that when he was 8 his teacher asked the class to write what they wanted to be when they grew up. David wrote ‘I want to be a girl’. Six years later he is desperately trying to find the courage to tell his parents, while conducting anxious inspections of his body for the changes of puberty. School starts for another year, bringing with it Leo, the new kid. Leo acts as the second narrator. Leo has his own secret which has led to him moving school and which, it seems, everyone at school is prepared to speculate on.

Having both David and Leo narrate the story works well as the characters are in contrast with one another and the friendship that forms between them manages to be simultaneously unlikely and utterly believable. Leo is certainly the stronger, more richly developed character with a wry sense of humour and plenty of wit. At times I felt David's interactions, especially with his best friends, were a little stereotypical and lacking in depth.

That said, there is plenty to like about all the characters and I laughed aloud on more than one occasion.

David makes reference early on to the fact that his life refuses to conform to the plot line of a ‘perky teenage movie’. Williamson plays with this familiar trope cleverly and manages to avoid delivering a saccharine ending while still rewarding the reader. Williamson wants us to realise that there is no art to being ‘normal’ because there is no such thing. This warm and funny novel adds to the growing body of work that tells us all that this is okay.

